# Importance of revealing a rare case of breast cancer in a female to male transsexual after bilateral mastectomy

**DOI:** 10.1186/1477-7819-10-280

**Published:** 2012-12-28

**Authors:** Dejan V Nikolic, Miroslav L Djordjevic, Miroslav Granic, Aleksandra T Nikolic, Violeta V Stanimirovic, Darko Zdravkovic, Svetlana Jelic

**Affiliations:** 1 Faculty of Medicine, University of Belgrade, Dr.Subotica 8, 11000, Belgrade, Serbia; 2 University Medical Center “Bezanijska kosa”, Bezanijska kosa bb, 10080, Belgrade, Serbia; 3 Dedinje Cardiovascular Institute, Heroja Milana Tepica 1a, 11000, Belgrade, Serbia; 4 Medicines and Medical Devices Agency of Serbia, Vojvode Stepe 458, 11221, Belgrade, Serbia; 5 Department of Oncosurgery, Clinic for Oncology, University Medical Center “Bezanijska kosa”, 11080, Belgrade, Serbia

**Keywords:** Female to male surgery, Mastectomy, Breast cancer

## Abstract

The incidence of breast carcinoma following prophylactic mastectomy is probably less than 2%. We present a 43-year-old female to male transsexual who developed breast cancer 1 year after bilateral nipple- sparing subcutaneous mastectomy as part of female to male gender reassignment surgery. In addition to gender reassignment surgery, total abdominal hysterectomy with bilateral salpingo-oophorectomy (to avoid the patient from entering menopause and to eliminate any subsequent risk of iatrogenic endometrial carcinoma), colpocleisys, metoidioplasty, phalloplasty, urethroplasty together with scrotoplasty/placement of testicular prosthesis and perineoplasty were also performed. Before the sex change surgery, the following diagnostic procedures were performed: breast ultrasound and mammography (which were normal), lung radiography (also normal) together with abdominal ultrasound examination, biochemical analysis of the blood and hormonal status.

According to medical literature, in the last 50 years only three papers have been published with four cases of breast cancer in transsexual female to male patients. All hormonal pathways included in this complex hormonal and surgical procedure of transgender surgery have important implications for women undergoing prophylactic mastectomy because of a high risk of possible breast cancer.

## Background

The incidence of breast carcinoma after prophylactic mastectomy is probably less than 2% [1]. Several studies such as those by Pennisi and Capozzi [2] and by Woods [3] have been conducted, where only few patients, from more than 1,000 patients included in the study (prophylactic subcutaneous mastectomy), developed breast cancer after years of follow-up (incidence rate 0.6%). However, one of the major concerns about nipple sparing mastectomy is the persistent risk of breast cancer development when this is used for prophylaxis, with much controversy about the safety of these procedures from an oncological point of view [4,5]. At present there are no randomized studies on the effects of long-term testosterone use on breast cancer risk. In a 20-year follow-up study of 110 female to male transsexuals in Serbia, there were none with breast carcinoma [6].

## Case presentation

A female to male transsexual, who underwent complex sex reassignment surgery after bilateral subcutaneous nipple sparing mastectomy (weight of removed breast tissue: left breast tissue, 275 g; right breast tissue, 295 g), presented 1 year after surgery with a painless left areoral mass. Samples of the left and right breast tissues sent for pathological analysis following subcutaneous mastectomy were benign.

The patient was 42 years old at the time the breast cancer was discovered, and 41 years old at the time of transgender surgery. The patient started testosterone therapy 18 months prior to sex reassignment (250 mg intramuscularly every 2 weeks). After the sex reassignment surgery, he received 250mg testosterone every 17 days for the next 12 months. At the time of sex change surgery, the patient was premenopausal.

The patient did not have any relatives with a history of any type of cancer. Family history of breast cancer and ovarian cancer in first-degree or second-degree relatives was thus negative, with no prior breast biopsies in clinical history, and therefore he was not a high-risk patient for breast cancer. BRCA1 and BRCA2 testing were not performed.

A routine chest X-ray image revealed an areolar mass on the left-hand side with N2 axillary node status, as well as lung metastases. Metastases in both lungs were confirmed by multislice spiral computed tomography. The classification, according to the American Joint Committee on Cancer, was T2N2M1, stage IV. Liver function tests and tumor markers (Ca 15–3) were normal.

Hormone profile studies confirmed satisfactory androgen replacement (normal testosterone with **luteinizing hormone** and **follicle-stimulating hormone** suppression.

A neoadjuvant therapy (fluorouracil, adriamycin and cyclophosphamide) 3-week protocol was administered to the patient, for six cycles. Owing to shrinkage of the tumor mass, radical mastectomy together with axillary dissection was performed after neoadjuvant therapy was finished. Trastuzumab (6 mg/kg intravenous infusion over 30 to 90 minutes every 3 weeks) was administered to the patient in combination with paclitaxel (80 mg/m2 every week) as adjuvant chemotherapy. Both drugs were administered to the patient after six cycles of fluorouracil, adriamycin and cyclophosphamide and radical breast surgery.

## Results

Histological examination of the breast tumor showed invasive ductal carcinoma of breast tissue and metastasis of invasive ductal carcinoma in the lymph nodes of the left axilla (12+/13),while the lymph nodes of the right axilla were negative for metastasis. Estrogen and progesterone receptors were negative although the Her 2/neu receptor was positive (60% of the tumor cells were 3+). Receptors for Ki-67 (50%) were positive. Androgen receptors (ARs) in breast cancer were also positive (2%).

Assessment of the tumor response following neoadjuvant therapy was good, with shrinkage of the primary tumor in the left breast from 63mm×54mm×50mm to 40mm×40mm×40mm.

For evaluation, a scintigraphy was performed along with four multislice spiral computed tomography scans (performed quarterly). No liver or bone metastases were revealed. Prior to neoadjuvant therapy, lung metastases were present in both the left and right lungs, with a maximum diameter of 11mm. Following neoadjuvant therapy these metastases were found to be smaller, with a maximum diameter of 9mm. After radical mastectomy and axillary dissection and 2 months after the start of treatment with trastuzumab and paclitaxel, only one metastasis with a diameter of 5mm was observed in the right lung on multislice spiral computed tomography of the thorax and abdomen. There were no metastases on other parts of the body.

## Discussion

The incidence of breast carcinoma after prophylactic mastectomy is probably less than 2% [1]. Several studies such as those by Pennisi and Capozzi [2] and by Woods [3] have been conducted, where only a few patients, from more than 1,000 patients included in the study (prophylactic subcutaneous mastectomy), developed breast cancer after years of follow-up (incidence rate 0.6%). However, one of the major concerns about nipple sparing mastectomy is the persistent risk of breast cancer development when this is used for prophylaxis, with much controversy about the safety of these procedures from an oncological point of view [4,5]. At present there are no randomized studies on the effects of long-term testosterone use on breast cancer risk. In a 20-year follow-up study of 110 female to male transsexuals in Serbia, there were none with breast carcinoma [6].

Our presenting case of breast cancer and lung metastases in a female to male transsexual exposed to exogenous androgens is very rare and has not previously been described. Direct effects of androgens on inducing breast cell proliferation and cancer via the AR are biologically impossible. In our patient, androgen-positive receptors were found in only 2% of breast tumor cells. *In vitro* studies have shown an inhibitory effect of androgens on breast cell proliferation and growth [7]. Negative findings for estrogen and progesterone receptors open the question of whether there is any hormonal dependence, or genetically determined carcinogenesis, irrespective of testosterone therapy.

The role of elevated androgen levels and the AR expression in male breast cancer as well as in female breast cancer is still unclear [8,9].

Based on a few early articles by Grattarola [10], the androgen excess theory states that urinary androgen excretion and intratumoral estrogen receptor status confirm the existence of hormone-dependent disease and predict the clinical outcome from ovariectomy in patients with metastatic breast cancer.

Positive AR immunostaining was found in approximately 70% of invasive female breast carcinomas and in a significant number of triple-negative tumors [11].

A recent study of case records for 1,849 patients with breast cancer revealed that positive AR immunostaining was inversely correlated with clinical stage, histological grade and mitotical score. Positive AR immunostaining was therefore associated with less aggressive tumors [12].

Standard therapy with antiestrogens and antiaromatase drugs is effective against increased estrogen production but quite ineffective against androgen excess. Additional therapy in these cases might be needed as well as determination of the origin (ovarian or adrenal) of the androgen excess in the particular patient. Ovariectomy (surgical, radiological or medical) would be indicated if the excess originates from the ovaries, while sulfatase inhibitors would be indicated in patients with adrenal source of androgen excess [9].

The Surveillance, Epidemiology and End Results cancer registry,which includes more than 2,000 male patients, has highlighted the fact that 93.7% of male breast cancers were ductal or unclassified, while 2.6% were papillary, 1.8% were mucinous and only 1.5% were lobular [13]. Breast cancers in men are significantly more likely to express hormone receptors than cancers in the female breast [8,13]. As much as 81% of male breast cancers express the progesterone receptor, and even 90% of them express the estrogen receptor. Knowing this, adjuvant hormonal therapy (including progestins, androgens, steroids, aminoglutethamide, estrogens, letrozole) has an important role in the treatment of these patients. However, literature data report AR expression in 34 to 95% of male breast cancer with no clear association with its prognosis [8]. Mutations in the AR gene have been reported in male patients with breast cancer [14], but again no causal association could be demonstrated.

It would be even more difficult to hypothesize on the role of androgen excess and the AR expression in the evolution of breast cancer in female to male transsexual patients due to the small number of such cases reported in literature. However, for those clinicians who deal with these patients, it is important to bear in mind all of the complex relationships between AR expression in breast cancer and other steroid receptors and growth factors.

There are certain dilemmas that must be addressed. In spite of the fact that the excised glandular tissue was pathologically benign, were the diagnostic procedures performed before sex reassignment surgery in our case insufficiently precise or insufficiently reliable, so that breast cancer had not been revealed in time, or was androgen supplementation the trigger for activation of invasive ductal carcinoma of the breast and potential high-speed malignancy of breast cancer, resulting in metastases in both lungs within a very short time?

According to medical literature, in the last 50 years there have been only three papers with four cases of breast cancer in transsexual female to male patients [15-17]. The first case, a 33-year-old female to male transsexual who developed breast cancer 10 years after cosmetic bilateral subcutaneous mastectomy and nipple reimplantation, was described in an article in *Breast*[15]. The following two patients mentioned in *Clinical Breast* were breast cancers diagnosed in two female to male transsexuals who had been treated with a supraphysiological doses of testosterone [16]. In addition, Gooren reported a single case of breast cancer in a female to male transsexual receiving testosterone hormone replacement therapy [17].

One should point out that the regularly performed bilateral removal of breast tissue including the nipple–areola complex, axillary tail and pectoral fascia was probably insufficient, and that breast cancer occurred in the residual breast tissue of the transsexual patient. However, it is very hard to speculate whether the patient could have developed breast cancer after oophorectomy with androgen supplementation if a total mastectomy had been performed.

As well as demonstrating the complex and poorly understood hormonal influences involved in the etiology of breast cancer, our patient’s management raised some important clinical issues. In this study the potential causative role of androgen replacement in breast malignancy, and the benefits, risks and safety of such treatment in breast cancer survivors, were discussed. The protection afforded to high-risk women undergoing prophylactic mastectomy is reviewed and the optimal hormonal management of this case was considered.

## Conclusion

This rare and very interesting case demonstrates that, in spite of the involvement of different types of specialists, such as surgeons, oncosurgeons, gynecologists and endocrinologists, very complex procedures of sex reassignment surgery can sometimes result in possible malignant disease escaping medical control.

Revealing and publishing cases such as this serves to raise awareness among doctors when starting the requested sex change process in these surgically and endocrinologically very complicated patients. The case also shows a need for change in clinical practice, to include magnetic resonance imaging prior to prophylactic mastectomy as a significantly more sensitive method for revealing invasive breast tumors [18].

## Abbreviations

AR: Androgen receptor.

## Competing interests

The authors declare that they have no competing interests.

## Authors’ contributions

ND: Participated in preoperative and postoperative surgical and chemotherapy following of the patient, carried out the molecular genetic studies, writing the manuscript, participated in the sequence alignment, corrected the manuscript in final draft. DM: Did the transgender surgery, writing the manuscript, participated in the sequence alignment, corrected the manuscript in final draft. GM: Did the removal of the cancer of the breast, carried out the molecular genetic studies, participated in the sequence alignment. NA: Did the checkups of the patient, carried out the molecular genetic studies, writing the manuscript, participated in the sequence alignment, corrected the manuscript in final draft. SV: Made major instructions according to the chemotherapy, carried out the molecular genetic studies, writing the manuscript, participated in the sequence alignment, corrected the manuscript in final draft. ZD: Followed the patient in preoperative and postoperative period, writing the manuscript, participated in the sequence alignment, corrected the manuscript in final draft. JS: carried out the molecular genetic studies, writing the manuscript, participated in the sequence alignment, corrected the manuscript in final draft. All authors read and approved the final manuscript.
